# Scientific user requirements for a herbarium data portal

**DOI:** 10.3897/phytokeys.78.10936

**Published:** 2017-03-24

**Authors:** Jorick Vissers, Frederik Van den Bosch, Ann Bogaerts, Christine Cocquyt, Jérôme Degreef, Denis Diagre, Myriam de Haan, Sofie De Smedt, Henry Engledow, Damien Ertz, Régine Fabri, Sandrine Godefroid, Nicole Hanquart, Patricia Mergen, Anne Ronse, Marc Sosef, Tariq Stévart, Piet Stoffelen, Sonia Vanderhoeven, Quentin Groom

**Affiliations:** 1 Clockwork, Ordina, Blarenberglaan 3B, B-2800 Mechelen, Belgium; 2 Botanic Garden Meise, Nieuwelaan 38, 1860 Meise, Belgium; 3 Fédération Wallonie-Bruxelles, Service Général de l’Enseignement supérieur et de la Recherche scientifique; 4 Université libre de Bruxelles (ULB); 5 Royal Museum for Central Africa, Leuvensesteenweg 13, 3080 Tervuren, Belgium; 6 Missouri Botanical Garden; 7 Belgian Biodiversity Platform, Belgian Science Policy Office (BELSPO), Avenue Louise 231, B-1050 Brussels, Belgium

**Keywords:** Botanic garden, collections, database, data sharing, digitization, science infrastructure

## Abstract

The digitization of herbaria and their online access will greatly facilitate access to plant collections around the world. This will improve the efficiency of taxonomy and help reduce inequalities between scientists. The Botanic Garden Meise, Belgium, is currently digitizing 1.2 million specimens including label data. In this paper we describe the user requirements analysis conducted for a new herbarium web portal. The aim was to identify the required functionality, but also to assist in the prioritization of software development and data acquisition. The Garden conducted the analysis in cooperation with Clockwork, the digital engagement agency of Ordina. Using a series of interactive interviews, potential users were consulted from universities, research institutions, science-policy initiatives and the Botanic Garden Meise. Although digital herbarium data have many potential stakeholders, we focused on the needs of taxonomists, ecologists and historians, who are currently the primary users of the Meise herbarium data portal. The three categories of user have similar needs, all wanted as much specimen data as possible, and for those data, to be interlinked with other digital resources within and outside the Garden. Many users wanted an interactive system that they could comment on, or correct online, particularly if such corrections and annotations could be used to rank the reliability of data. Many requirements depend on the quality of the digitized data associated with each specimen. The essential data fields are the taxonomic name; geographic location; country; collection date; collector name and collection number. Also all researchers valued linkage between biodiversity literature and specimens. Nevertheless, to verify digitized data the researchers still want access to high quality images, even if fully transcribed label information is provided. The only major point of disagreement is the level of access users should have and what they should be allowed to do with the data and images. Not all of the user requirements are feasible given the current technical and regulatory landscape, however, the potential of these suggestions is discussed. Currently, there is no off-the-shelf solution to satisfy all these user requirements, but the intention of this paper is to guide other herbaria who are prioritising their investment in digitization and online web functionality.

## Introduction

A quiet revolution is happening in the way herbarium specimens are being accessed and used. Botanic gardens, museums and universities, all over the world, are digitally imaging herbarium specimens, transcribing their details while geolocating their origin (Baird 2010, Blagoderov et al. 2012, Van Oever and Gofferjé 2012, Tegelberg et al. 2012, Heerlien et al. 2015, Thiers et al. 2016). These activities will radically improve access to herbarium specimens and will result in many benefits for science. For example, it will empower botanists from southern countries by giving them access to historical herbaria in the north; it will provide the data held on these specimens for research and conservation and it will improve the efficiency of plant taxonomy. By giving a herbarium a virtual existence, we can radically change the way herbarium collections are used, increasing the versatility of herbarium specimen data, and even open them up to an audience who may previously been unaware of their existence.

Improving access to biological collections is a policy goal of many governments and organizations. For example, Article 17 of the Convention on Biodiversity focuses on the exchange of information, and target 19 of the Aichi Biodiversity Targets relates to biodiversity knowledge exchange (Convention on Biological Diversity 2010). Many institutions and individuals support these changes and some have ratified this by signing the Bouchout Declaration (pro-iBiosphere Consortium 2014). This declaration promotes open access to biodiversity data through the use of information technology infrastructure, standards and protocols.

In addition to their traditional role in plant taxonomy, improved access promotes new lines of research and applications for herbarium specimens. Such data can be used to monitor environmental changes, such as changes of plant phenology that result from climate warming (Vellend 2013, Rawal et al. 2015). These data are also essential to the rapidly growing field of species distribution modelling (ecological niche modelling). The data can also be used to study phytogeography, biological invasions and range shifts (Barney 2006, Lavoie 2013, Groom 2015). In regions where the available botanical knowledge is poorly documented, herbarium data can represent a valuable source of information on alien species (Fuentes et al. 2013). In community ecology, they can even be used to identify changes in community composition (Cocquyt et al. 2008). They can also be used in historical research, for example by recreating the prosopographical networks of botanical exchange (Groom et al. 2014). This has recently been demonstrated by a paper on the Belgian botanist Ernest Sonnet (1840-1901) based upon digitalized herbarium sheets kept in the Herbarium of Rio de Janeiro (Hanquart et al. 2016).

The Botanic Garden Meise holds around 3.5 million herbarium specimens from around the world, with important historical collections, and a clear focus on Central Africa and Latin America, as well as additional significant collections from Belgium and Europe. In 2002, the Garden started imaging and cataloguing its collections with two small pilot studies within the EU funded framework of the European Network for Biodiversity Information: namely The Albertine Rift Project (Stoffelen et al. 2005b) and the Martius’ Flora Brasiliensis project (Stoffelen et al. 2005a). The digitization effort was intensified by projects such as the African Plants Initiative (Smith 2004), the Latin American Plants Initiative and the Global Plants Initiative, all funded by the Mellon Foundation (Ryan 2013), and by other digitalisation projects funded by the Belgian Science Policy Office through the Belgian Biodiversity Platform (Stoffelen et al. 2009), the European Commission and the Belgian Science Policy Office (Cocquyt et al. 2007, Botanic Garden Meise 2014). Progress has been slow due to limited resources and conflicting priorities. Previous scanning projects have focussed on the type specimens (ca. 55,000 specimens) and selected historical collections. Digitization of the whole herbarium was not deemed feasible in the foreseen future. However, in 2015 the Flemish Community funded a project to digitize all of the Garden’s African and Belgian collections and improve the infrastructure for photography and microscopy. This project is called *Digitale Ontsluiting Erfgoedcollecties* (DOE!), which translates to Digital Unlocking of Heritage Collections.

For the Garden this project presents many opportunities. It will update and extend the current digitalisation infrastructure, image storage and web portal. In addition, the project will raise the profile of the Garden; demonstrate the importance of the collections and make the general public more aware of the collection’s existence. It will also increase awareness of the Garden’s research initiatives and its relevance to conservation, science and society.

**Figure 1.**
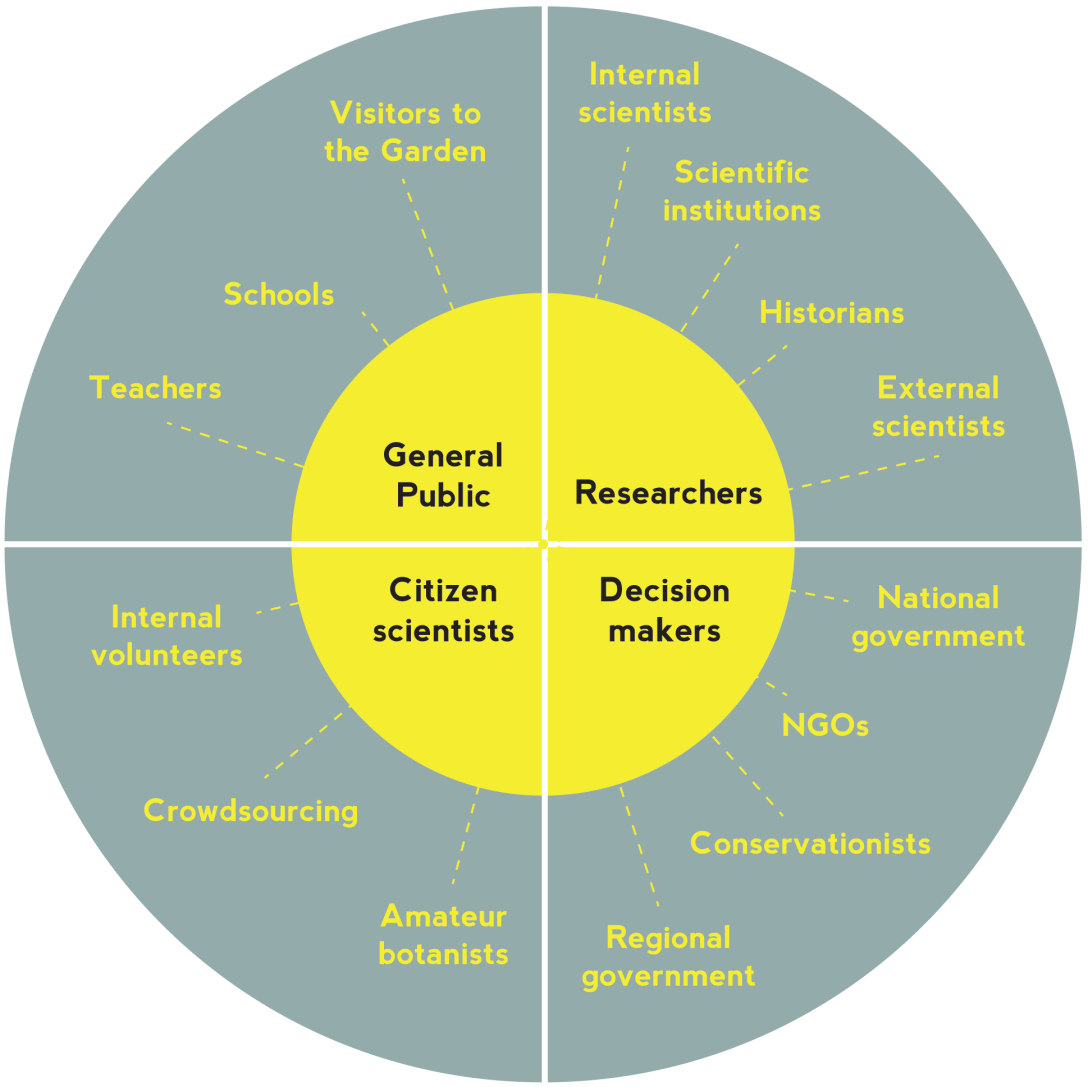
Stakeholders interacting with the Botanic Garden Meise and potentially using its data portal. The stakeholders prefixed by the words ‘internal’ refer to those that work at the Botanic Garden, whereas those referred to as ‘external’ refer to researchers in other institutions.

As part of the DOE! project, the Garden will update its herbarium portal. However, before redesigning the portal and making key decisions on data management, the Garden decided to conduct a user requirements analysis to establish the needs of scientists and other user groups and help prioritise investment. This prioritisation is necessary as funding is limited and we wish to fulfil the demands of the diverse users. Many different people and organizations interact with the Botanic Garden Meise and may access its data portal for different reasons (Fig. 1). Teachers and schools may use the herbarium portal to demonstrate the diversity of plant life and the role of specimens in research. Citizen scientists may use the portal for their personal interests and as an element of volunteer work. Also, decision makers may use the summarized data to guide management priorities and decisions. Nevertheless, traditionally the main users of herbarium specimens and their associated data are researchers. Therefore, we have concentrated our user requirements exercise on researchers who we considered to be the primary users, but we consider that improving usability and functionality for this group is likely to benefit all stakeholders. In the long term it is conceivable that different types of users could be directed to different views of the data tailored to their needs.

In parallel with the user requirements analysis, the Garden has also developed a data management plan to clarify its position on data management and access towards digital herbarium data. This plan highlights system requirements for the herbarium data portal, which may not be directly visible to the users, but are nevertheless important to support the accessibility and citability of data. The plan will also clarify issues of licensing and data embargoing that are needed to make it clear what the limitations are on the use of data.

Having completed the process of user requirement analyses, we considered that the insights gained should be shared with other herbaria and museums. To this end, we present here the process and outcomes of this user requirements analysis. We identify similarities and differences in user expectations. We also identify requirements that users considered to be important, and suggested they be prioritised.

The mass digitization of herbaria around the world presents enormous challenges and opportunities for science. Ultimately, its success will be judged on the impact these efforts have on scientific progress and on society in general. An important part of this effort is to empower users with the tools and data they need to make an impact.

## Methods

The Botanic Garden Meise contracted Clockwork, the digital engagement agency of Ordina, to conduct the user requirements analysis. Clockwork has extensive knowledge in the field of user experience and digital design and their lack of knowledge of botanical research was considered an asset as it helped to provide a fresh perspective on the user requirements for the herbarium portal.

### Preparatory phase

Before consulting stakeholders a small team, comprising staff of the Garden, met to identify potential stakeholders and decided on those to be consulted in a requirements analysis as discussed in the introduction (Fig. 1). In this phase, researchers were divided into three groups based upon their domain of expertise: taxonomists, ecologists and historians, though, it is appreciated that other researchers, including sociologists and geneticists, may also use herbarium data on occasion.

The second step of the preparatory phase was a “market analysis” where Clockwork and a core team from the Garden conducted a survey of the online landscape of herbarium tools and resources. The information gathered at this phase was used to inform ourselves of the current state of the art, so that questions could be framed and the opinions of the stakeholders could be contextualized.

### Recruitment

Participants were recruited from scientists and historians of the Botanic Garden as well as from Belgian universities and scientific institutions. Externally, the recruitment was made by invitation in order to have some control on the representation of participants. Within the Garden, participants were recruited from a mixture of invited staff and self-selected volunteers. An effort was made to recruit from a diverse range of participants where scientific discipline, gender, language and origin were considered. Scientists were broadly recruited from within their fields, including those interested in tropical *versus* temperate regions to those studying vascular plants *versus* cryptogams. In total, 23 participants were recruited; 12 taxonomists; 7 ecologists and 4 historians. These included 10 women and 8 external researchers.

### Interview format

The approach was for pairs of participants with similar jobs to be interviewed together. Each pair was presented with different task-scenarios related to their daily work and for which they would apply information from the herbarium. These tasks were selected to be representative of the tasks of each type of scientist identified during the market analysis workshop (Table 1). Participants were paired based on their similar scientific backgrounds and interests, to avoid discussions arising from differing perspectives. Each pair had to choose one of the three task scenarios. They were then asked to create a task breakdown of how they would fulfil the task. The goal was to understand the processes that these scientists would routinely perform. The participants wrote each step of their task on individual colour-coded sticky notes and arranged them out in chronological order. The aim of the next step was to create a list of types of data or information that scientists would be looking for when being faced with the specific scenario. The participants were asked to list what types of data or information they would be looking for at each step of their task breakdown. Every type of data or information was written down on an individual sticky note and placed beneath the related step of the task breakdown. Once the lists of data and information had been created, the next step was to investigate what sources and platforms they would use to find these data and information. The participants were asked to list all sources and platforms that they would use to gather the previously listed data and information at each step of their process. They were explicitly triggered to think both in terms of digital and non-digital sources and platforms. All listed platforms and sources were written down on individual sticky notes and placed beneath the related data type.

**Table 1.** Task scenarios presented to participants in the user requirements interviews.

Historical based scientists: 1. Write a biography on a collector called Joseph Bequaert, on his voyages, taxonomic interests and the people he worked with. 2. Contrast the traditional uses of the genus Solanum in Africa and South America, then comment on the impact of modern Solanaceous introductions to traditional agriculture. 3. The garden has received a large collection of photographs on glass plates. There are some limited details that come with the collection, but you would like to improve the metadata associated with each image.
Ecological based scientists: 4. You need to start gathering data for a species distribution model of *Agrimonia eupatoria*. Where would you get the data and ensure that they are free from errors? 5. You need to create a red-list for Belgium. Look for the necessary data and determine the status. 6. An alien species is gradually moving northward in Europe. Before it has naturalized in Belgium you need to write an impact assessment for decision makers.
Taxonomic based scientists: 7. You find a specimen in another collection's herbarium. You can't read the signature but have the locality and the date. How would you figure out who the collector was? 8. You think you have discovered a new species in the herbarium collection. How would you verify that it has not already been described? 9. You are writing a revision of a large genus. You need to create a distribution map of each species.

After listing the platforms and sources that participants would use, we focused on the reasons why they choose these over other sources and platforms. They received a template for each source or platform they listed, up to a maximum of three. On this template, participants were asked to describe their experience of the source or platform; they listed its key functionalities, its strengths, its weaknesses and if there was something that could be improved or is missing.

Then, the focus moved to the current virtual herbarium of the Botanic Garden Meise in order to get an overview of its strengths and weaknesses according to the participants.

Before the participants were able to access and explore the current virtual herbarium, they were asked three questions:

*Did you know that you can access a part of the Garden’s herbarium collection through the virtual herbarium on the website*?

*Have you ever accessed or used the virtual herbarium before? If so, what was your reason for using the virtual herbarium*?

*Did it meet your expectations? If not, why not*?

During the exploration of the current virtual herbarium, they were also asked:

*When you look back at the task that we worked on during this interview, do you think you could have used this virtual herbarium to fulfil one of your steps*?

*Why, or why not? What is missing in order to help you fulfil your task*?

The final step of the interview focused on consolidating the input that was given by the participants during the previous steps. The goal of this step was to stimulate the participants to convert their feedback on external platforms as well as the Garden’s virtual herbarium into concrete requirements for the future virtual herbarium portal.

The key question that was asked to the participants at the end of the interview was “*My ideal virtual herbarium should...*”. Participants wrote down elements, functionalities, integrations, data-links, etc., they deemed to be crucial for the future virtual herbarium.

### Analysis of the outcome of the interviews

The interviews were conducted by two user experience researchers both with backgrounds in qualitative data analysis. One of the user experience researchers moderated the interview, while the other noted the feedback provided by each participant.

**Figure 2.**
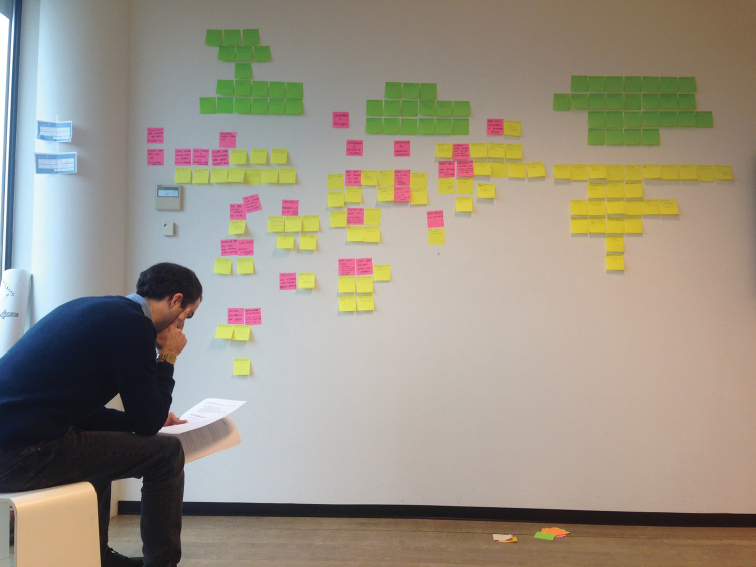
A user experience researcher using affinity diagramming to cluster user requirements from the results of the interviews.

The interview notes were then enriched and digitized. The feedback of the participants was initially analysed by reading each comment and interpreting the underlying meaning. Affinity diagramming was used, as a qualitative content analysis method, over two iterations to establish core themes and then establish sub-themes (Kawakita 1991). These themes were linked together and structured on the level of each type of researcher. After creating these structured lists, the insights and needs were mapped using of colour-coded sticky notes that distinguished requirements that were related to data and requirements related to functionality (Fig. 2).

The final step in the analysis approach consisted of manual clustering of closely related needs and insights. These clusters helped to identify overlap among the requirements that were proposed by the different types of researcher. These overlaps served as the basis for defining common user requirements.

## Results

A detailed list of all user requirements is provided in the supplementary data, but have been summarized below. An important distinction was between the requirements for data and functionality. This is pertinent because user requirements for functionality can be addressed in portal development, but data requirements need to be address in long term digitization strategy.

### Data requirements

The majority of researchers that participated in the interviews explicitly mentioned the need for as much data from a specimen as possible. They felt that the quality of their analysis was strongly dependent on the amount of data that might be interrelated and analysed. The most important source of these data is the specimen itself. Taxonomists, historians and ecologists highly valued the ability to consult the physical specimen in the herbarium or failing that, a high quality zoomable image. The information that can be derived from these specimens goes beyond the data that is written on the labels. Taxonomists for example study the physical specimen anatomically, microscopically and genetically, while historians derive information from details such as the layout of the label, the handwriting, and even the type of paper used to display the specimen and the label. Finally, ecologists are interested in field notes that the collector added to the specimen, as these often contain information about the habitat, substrate and other environmental data. The detailed information on a herbarium specimen includes its label information, annotations and even the way it is mounted. Even when scientists are provided with a virtual herbarium they will often need to consult the physical specimen for further details.

**Figure 3.**
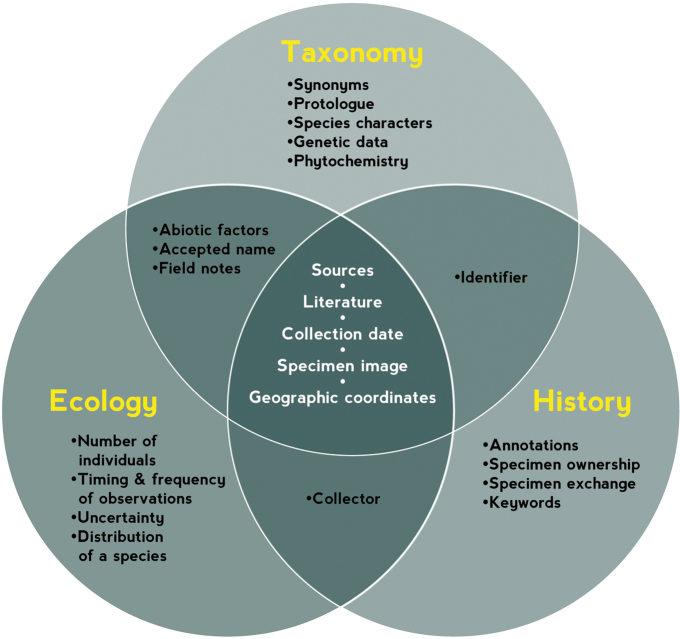
A summary of the data elements mentioned by the different researcher types, showing which data elements researchers had in common and which were unique. This does not mean that any particular data element is not of interest to another group, only that it did not arise in the series of interviews. Details of these data elements can be found in the supplementary information. The full list of common data elements is listed in Table 2.

The common data elements identified across the different researcher categories are listed in Table 2 and subdivided by their importance from the researchers’ perspective. Also summarized are the shared data elements that different researcher types mentioned (Fig. 3). There is a clear set of common data elements shared by all research groups. An even greater overlap exists between data elements of ecologists and taxonomists. Among the data elements mentioned uniquely, historians mentioned those related to the human creators and curators of specimens, such as details on specimen exchanges. Taxonomists have a specific interest in elements related to botanical nomenclature (type material), while ecologists feature data related to populations and statistics. Nevertheless, it is important to highlight the common elements related to the provenance, date, citation and geographic coordinates. Also, all groups wanted access to the original image.

**Table 2.** Common data elements identified across the different researcher categories as being important to their work.

Key data elements	‘nice to have’
Current name and classification of the specimen	Abiotic factors related to the specimen
The location where it was collected (ideally coordinates)	Information about the habitat of the collected specimen
Country	Ecological information on the location where it was collected
Date of collecting	Information on meteorology
Name of the collector	Description of characteristics on both macro- and micro level
Collection number given by collector	Being able to measure the length of leafs, flowers, … on the high resolution image via an intuitive tool that makes it able to draw lines
High resolution photo of the physical specimen (to get access to the metadata on the label that was not digitized in the database)	

### Linking databases

Throughout the interviews, it became clear that the requirements for data go beyond the data that can be accessed directly *via* the herbarium. When the participants were asked to describe their ideal virtual herbarium, all three types of researcher repeatedly mentioned the value of creating links between data in different databases within as well as outside the Botanic Garden. The heterogeneity of data sources increases the effort required to find relevant data and also risks data being overlooked.

### Internal databases

The Garden’s internal databases include those of the library, preserved plant collections, seed bank, living plant collection and photograph collections. Connections between these databases would facilitate research and simplify access to resources. For example, historians strive to reconstruct the sources of collected knowledge and data by looking for links between people, specimens, locations and collections. They would like links between herbarium data, gazetteers, biographies of collectors and the library catalogue. Taxonomists would like data and pictures of the Garden’s living collections alongside the dried specimens from the herbarium. Ecologists would like links to field notes of the collector to provide a deeper understanding of the habitat, plant stage, and other factors related to the specimen.

### External databases

The main reason to link to external databases and platforms is to facilitate finding relevant literature and additional data. Suggestions for useful linkages included: the Biodiversity Heritage Library (www.biodiversitylibrary.org) for literature; JSTOR (www.jstor.org) for type specimens; the Global Biodiversity Information Facility (www.gbif.org) (GBIF) for plant distribution; and, nomenclatural information from the African Plant Database (www.ville-ge.ch/musinfo/bd/cjb/africa); The Plant List (www.theplantlist.org) and IPNI (www.ipni.org/index.html). Other sorts of data and information can be provided through links to botanical illustrations, photographs and maps (historic and modern). Links to other herbaria, particularly to duplicate specimens, were considered important. This would assist curation and verification of material through taxonomic revisions in other herbaria. Scientists ideally would like a single shared and interactive portal for all herbarium specimen information.

### Interlinking names

Taxonomists attach importance to the correct identification and correct names. However, not the only scientists stressed this importance. It was suggested that the integration of a simple nomenclatural overview for each specimen would be valuable where the current name, related synonyms and the common names are mentioned.

This list could be used to search for and collect relevant data even if the accepted name is different in other databases. Using a smart search engine that shows all relevant data, material and literature for a particular taxon would be very valuable, by reducing the risk of overlooking data due to synonymy.

Finally, all three types of researcher expressed value in being able to track name changes on a specimen.

### From a complex query to a usable search function

Many researchers within the Garden mentioned that they had to request complex data extracts from the curator. Improving querying and extraction of data would save curatorial staff and scientists’ time. Participants were enthusiastic about the idea of accessing the current virtual herbarium *via* a user-friendly online interface. Even though the current web portal lacks some functionality, all of the participants appreciated its speed and liked being able to search for data with a few clicks instead of typing complex search queries.

A specific functionality requested by the taxonomists was the ability to define a bounding box or polygon to select specimens from an area. This would help them plan field trips, but also could help them judge the ecological conditions of the area. It might also be useful for creating simple summaries such as a checklist of trees or endangered species of an area.

All of the participants wanted to be able to filter and sort the results of their queries. After which they should be able to download the dataset in a usable, spreadsheet compatible format. They felt that herbaria are public sources of data and that the virtual herbarium should support them to retrieve the right data. However, the actual analysis of these datasets should be conducted by the scientists outside of the virtual herbarium environment.

### Data centralization

The scientists that participated in the interviews were, in principle, open to the idea of moving their personal datasets to a central database. The main reason why they create local datasets is to be able to work with their data within a comparatively simple environment, while the central database is often too rigid.

The idea of using the online portal as a tool to insert data centrally was received quite positively by the different types of researcher. “*We have to digitize our data somewhere, so we might as well do it directly in the central database and get the opportunity to relate our data with other data and extract it in a usable format for analysis*”.

Furthermore, it would be convenient to link materials, photographs, data, etc. stored in a central database. Firstly, this database would enable the scientists to access their data and other material remotely. Secondly, a centrally managed database would lower the risk of catastrophic loss. Finally, this database could take care of standardization of their data, including taxon names, collector names, country names, etc.

### Validation process for added or modified data

Researchers pointed out that editing data directly could add errors if editing is unrestricted. Opening up the system to uncontrolled data input could reduce the quality of information, potentially harming the data significantly. To balance the reliability of the herbarium data, openness should be met by a need for transparency of how the data are derived.

In the researchers’ opinion data editing rights should only be granted to approved users through username and password control. But, even then, such edits should always go through a review process before overwriting the existing data. Edits would be sent to a validator or data manager, who reviews them. In the meantime, pending adjustments could already be made visible to other users while they are still under consideration. As such, visitors can already benefit from the new data, knowing that it is provisional and not yet validated.

In order to streamline the process of data validation, researchers should be able to take on the role of the validator. As a validator they could subscribe to updates about changes to specific parts of the database linked to their field of expertise. This would enable them to keep track of what happens to data connected to their own work, and also bring their expertise into the validation process.

The need for transparency also reflects on what researchers expect to see after the validation process. Based on the interviews the outcome of this validation process should be made visible via a data history feature. Users should be able to track back what happened to specific data in the past. Incorporating a history of changes would help researchers understand the evolution of data, which in turn could lead to more informed decisions on future modifications. A number of taxonomists and historians mentioned that this history of data could even serve as a starting point for future research projects. The history of data described above would provide transparency on the origin of data, which in turn provides an indication of its reliability. Several participants also suggested adding a clearly marked reliability factor to validated data.

### Data access

There was a remarkable difference of opinion among the scientists on whether data should be open or partially closed to external users. One of the main reasons for closing data was the fear that external scientists would “*steal*” the data, ideas and expertise and publish on it first. This concern is particularly present in the case of new specimens, collected during recent expeditions, or for specimens currently being used in research projects.

The idea of locking away certain specimens was mentioned a few times. Some scientists want to be able to embargo specimen data for a period or the duration of a project. Others believe it would suffice to make some data hidden from external users. In this case, users with an internal account would still be able to see all data. Data could be hidden by simply marking information ‘internal only’.

In contrast, there are researchers who believed hiding specimen data goes against the Garden’s role as a public institution. They felt strongly that data should be shared with all those working on biodiversity, regardless of whether the person works inside or outside the Garden. Only for newly collected data do they agree the need for a temporary embargo.

The opinion was also divided about being able to download data sets. Some researchers were opposed to making this option easy for external users. For them, it should be mandatory for external people to identify themselves before being able to download data sets from the platform.

## Discussion

There are several methods to produce user requirements, including prototyping, observing users, analysing pre-existing systems, focus groups and surveys. We engaged an external agency to leverage their expertise in creating user requirements with our botanical expertise. In selecting a suitable subcontractor the methodology was an important criterion as we wanted a consultative approach so that the stakeholders at the Botanic Garden were engaged with the process. Nevertheless, owing to the time and costs of such an approach we did have to limit our investigation to researchers living in Belgium. A useful follow-up to this study would to be to repeat this exercise in a tropical country where the benefits of data repatriation could be analysed.

The user requirements exercise demonstrated, to our own surprise, that researchers of different disciplines had similar needs. Both in terms of their data requirements and the functionality for a web portal. Furthermore, delivering all these requirements would be a significant challenge, even for large institutions with sufficient IT resources. It is clear that development has to be prioritised and requirements need to be rated on their cost-effectiveness.

Transcription of label information is one of the most time consuming aspects to digitization. Furthermore, geolocating specimens considerably increases the skill and effort required. Ideally all specimens would be transcribed, catalogued, photographed and geolocated, but decisions need to be made on the best way to achieve this, both from the perspective of cost and user needs. Is it better to have a little data from every specimen or all the data from some of the specimens? Users had broad data requirements, wanting as much of the label information as possible. So for most users it is better to transcribe the whole label of fewer specimens. This also makes the transcribed data more useful for a wide variety of research topics. However, if minimal data were recorded that enable users to find specimens comparatively easily, the image could be consulted directly to gain the additional information. So having an image available, even without much of its metadata, will support full transcription in the future and is a cost effective way to disseminate information. Users anticipate consulting individual specimens even where the same digitized data are available online. Given the limitations on the rate of transcription, the most appropriate strategy would be to consult researchers as to which specimens to prioritise for transcription, but then completely transcribe the label information on those specimens.

The importance of linking herbarium data to internal and external databases was a requirement of all users. For example, in the case of ecology, linking taxonomy to trait data can be used to assign taxa to functional groups and facilitate modelling of ecosystems based upon these functions (Pendry et al. 2007). Such analyses can be used to model the geographic distributions of functional groups, but also predict the impact of environmental changes on these groups and the ecosystem services they provide (Engemann et al. 2016).

A technical requirement related to linking is the need to ensure persistence of these links. Herbarium portals therefore need to provide a permanent URI to a specimen (Hyam et al. 2012, Hagedorn et al. 2013). Such stable identifiers also provide a method to cite specimens from a publication. This is also an important aspect in the FAIR Guiding Principles for scientific data management and stewardship, which aim to ensure data are findable, accessible, interoperable and reusable (Wilkinson et al. 2016). These principles ensure that data are human and machine readable, but also that provenance is tracked to support scholarly citation. These are issues that can be addressed by an institutional data management plan.

Although it was not mentioned by the scientists, interlinking may reduce redundancy between databases and therefore reduce the curatorial effort of maintaining data such as taxonomic names and citations. Linking databases would make it possible to automate the process of updating names when a duplicate receives a new name in another herbarium. These links could also facilitate the exchange of georeferencing information and other details of the specimen. It will be necessary to make the origin of these data clear online, both to credit the sources and to give an indication of its reliability.

Linking of databases potentially brings together a large amount of complicated data that needs to be summarized succinctly. This need for data consolidation was a general requirement of participants. Currently, they often start from sources that consolidate data at the level of species and genus in a handy overview. Two good examples of platforms that provide such summaries are Tropicos (www.tropicos.org) and the GBIF (www.gbif.org). The types of data that are consolidated by these platforms consist of: an overview of names and their related literature; a hierarchical classification; the distribution of the taxon shown on a map; descriptions of the organism; links to other sources that contain related data; links to publications and images of preserved and living specimens.

From the requirements it is clear that it is impossible to separate the user requirements for an internal collections database and an online portal. For example, an online commenting system would either need a workflow to integrate these comments back into the main database, or the web portal would be just one view of the institution’s main database, with all the security and capacity implications that architecture would have. There are various competing systems for herbarium database systems including several bespoke solutions. Examples include BG-BASE, DaRWIN, DINA-Web, BRAHMS, JACQ and Specify. It is safe to say that none of these solutions provides all the user and system requirements detailed here. Certainly, the Botanic Garden Meise is not alone in the struggle to maintain legacy systems and create modern interfaces with obsolete technology. The lack of suitable alternatives eliminates the possibility of providing many user requirements with one simple software change. Rather it seems we must aim for incremental change, whilst trying to ensure these investments are at the same time future-proof for when new solutions become available. The best way to do this is to ensure that the data are maintained in standard formats and conform to standard controlled vocabularies.

One of the greatest concerns within the current information technology landscape in biology are orphaned data (Gibney and Van Noorden 2013). Scientists and institutions such as the Botanic Garden Meise house an enormous amount of data. However, much of these data are inaccessible due to scientists creating their own datasets and storing these locally on their computers instead of sharing them centrally. Scientists appreciate the advantages of moving their data sets and material to a centrally managed database. The main barrier to import these data sets into a central one is that scientists perceive the current database as too complex and rigid. Scientists prefer to use their own formats to structure data. They expect the platform to offer the ability to add data regardless of the rigid structure of the central database. One should question whether this is a valid requirement to support. There are numerous advantages for downstream users if data are conformant to community agreed standards and described clearly with metadata. If researchers were able to deposit non-standard data in repositories in whichever format, this may save them time, but would result in unusable data, not because they are lost, but because they are incomprehensible. A solution would be to facilitate scientists by providing software tools that maintain data simply, but also reward and mandate data archiving.

All researchers thought that feedback systems for data would be a valuable addition to an online portal. A good example of where feedback is used to effect is on the Encyclopedia of Life website (http://eol.org). Here, changes and comments are displayed on taxon pages. Such a system would satisfy the researchers’ requirement to annotate records with their comments. However, translating these annotations into corrections that can be applied to the master database is an administration challenge, due to difficulties of contentious decisions where it is difficult to judge the authority and priority of edits. A compromise could be to implement a review system, whereby users can rate entries in addition to commenting. In this way potentially problematic entries can be flagged for review. Yet, these problems only need to be resolved when a user wishes to use a datum.

The most contentious subject among scientists is whether and how data are shared. This is a subject of much debate within the research community (Reichman 2011, Groom et al. 2013, Ferguson 2014). In a survey of natural history collections, data sharing was the greatest barrier to digitization that was not related to funding and resources (Vollmar et al. 2010). Many authors have promoted the concept of open data and its advantages for science and society (Molloy 2011, Gibney and Van Noorden 2013, Van Noorden 2013, Groom et al. 2015). In Belgium, both the Federal and Flemish governments have open data policies for public data and open data is considered their default position (Vlaamse Regering 2011, Delafortrie and Springael 2015). These policies follow the European Union’s directive on the re-use of public sector information (Cox and Alemanno 2003). Furthermore, other public Flemish scientific institutions have adopted open data policies (Desmet et al. 2014, VLIZ 2016). Nevertheless, there is an inherent conflict of interests between the Garden’s role as a guardian of knowledge and as a research facility where the balance between these roles is still being determined. While the pros and cons of data openness will not be debated here, this argument underlines the importance of having a data management plan, as some of the user requirements can only be achieved using open data.

Most, if not all, of these user requirements will be familiar to curators, taxonomists and others who regularly work with herbarium data. Nevertheless, it is valuable to record these requirements for several reasons. We need to deliver as many of the requirements as possible, but also keep a record of our progress. Prioritisation is also critically important to make effective use of the available budget. Furthermore, it is useful to communicate our needs to other institutions because fulfilment of some of the user requirements requires cooperation and adoption of common standards by many institutions.

## Conclusion

Researchers have high expectations of biodiversity informatics, both for the software and the data that have been digitized. User requirements are similar for different types of researcher and we should prioritize access to core data fields in an easily searchable and useable format. Nevertheless, the most useful way to prioritize the transcription of label information is to work on data that is required immediately for research, but always transcribe the whole label data. Furthermore, though researchers appreciate simple access to digital images and data, they still value access to the original specimens.

## Supplementary materials

Supplementary material 1 Gathered needs per type of researcher Data type: form This dataset is made available under the Open Database License (http://opendatacommons.org/licenses/odbl/1.0/). The Open Database License (ODbL) is a license agreement intended to allow users to freely share, modify, and use this Dataset while maintaining this same freedom for others, provided that the original source and author(s) are credited.Jorick Vissers, Frederik Van den Bosch, Ann Bogaerts, Christine Cocquyt, Jérôme Degreef, Denis Diagre, Myriam de Haan, Sofie De Smedt, Henry Engledow, Damien Ertz, Régine Fabri, Sandrine Godefroid, Nicole Hanquart, Patricia Mergen, Anne Ronse, Marc Sosef, Tariq Stévart, Piet Stoffelen, Sonia Vanderhoeven, Quentin Groom
