# Supplementary material for: Scientific user requirements for a herbarium data portal
Source: PhytoKeys. 2017 Mar 24;(78):37–57. doi: 10.3897/phytokeys.78.10936 (PMC5543274; doi:10.3897/phytokeys.78.10936)
Supplement: Supplementary material 1 — Gathered needs per type of researcher [file phytokeys-78-e10936-s001.docx]

# Gathered needs per type of researcher

## Taxonomical based scientists

### Needs for data

The taxonomical based scientists mentioned the following types of data, when they were asked which information they needed in order to fulfil their task scenarios.

1. A complete list of all possible names of species (aggregated overview of the valid name, synonyms, and local names).
2. High-resolution photographic material of specimen (both on a macro- and microscopic level).
3. Descriptions of characteristics on a microscopic level.
4. Descriptions of characteristics on a macroscopic level.
5. The protologue of species.
6. All of the information that can be found on the label of a specimen (as detailed as possible).
7. The accepted name of the specimen.
8. Name of the collector and their collection number
9. Name of the identifier and the date of the identification act.
10. Location where the specimen was collected.
11. Country
12. Locality
13. Coordinates (together with geodetic system).
14. Date of collecting.
15. Ecological information (substrate, habitat, etc).
16. Further description of the specimen that was provided by the collector.
17. Field notes of the collector about a specific specimen.
18. Chemical data.
19. Genetic data, such as chromosome counts and DNA sequencing that has been conducted on the specimen.
20. Literature related to the taxon.

### Requirements of taxonomical based scientists

1. Supporting for the clustering of specimens into groups.
2. The more metadata the better.
3. All information that is present on a herbarium specimen needs to be digitized and accessible through the virtual herbarium.
4. Next to the higher plants, we also need access to smaller collections such as those of fungi and algae.
5. Access to the actual specimen will still be required for microscopic and genetic analysis.
6. Phenological information on the specimen, such as flowering stage at the moment of collection.
7. A handy tool to measure the dimensions of objects on the high resolution image.
8. Coordinates are useful and should be as precise as possible.
9. The system needs to consolidate data in a smart way. Information about the locations of specimens should be visualized on a map and can be enriched with ecological and climatic information about a region.
10. An overview at the level of species that consolidates the nomenclature, a protologue, images of living specimens and a visualized distribution on a map.
11. The search function should recommend related data.
12. Integration of gazetteers would make it possible to convert location names to coordinates in a smart way.
13. Linking data of internal and external databases:
14. Internal databases that should be linked to the herbarium:
    1. Living plant collections
    2. Photos of living specimens
    3. The seedbank
    4. The library
15. Interlinking the duplicates of specimens that were distributed to other herbaria, particularly to keep the names up-to-date, between institutions.
16. External databases that might be linked:
    1. Smart links to relevant literature and publications from the Biodiversity Heritage Library (http://www.biodiversitylibrary.org/).
    2. Virtual herbaria of other institutions to access to more specimens
    3. The International Plant Names Index (http://www.ipni.org/) supporting nomenclature.
17. Links between current names, synonyms, local names, so the system can make smart data recommendations. The system should make smart recommendations, so you are able to search for data by a part of a name.
18. A history of name changes on a specimen that shows who changed a name at what moment in time.
19. Being able to add a ‘name in progress’ to a specimen that has not been published yet.
20. Smart links between specimens (duplicates) that are located within other institutes. If one of the duplicates is given a different name, all other institutions should be informed.
21. Keeping control over data.
22. Limiting the accessibility of external scientists.
23. Downloading of datasets by external scientists should only be possible after identifying themselves.
24. Datasets are open for everyone, but it should be possible to mark what can be published and what should only be available for internal use.
25. Data history/track changes to see who made which changes to data in the past.
26. Being able to easily add data to the central database without having to use a rigid data structure.
27. It should be visible when data has been added, but not yet validated.
28. When adding data you should be able to mark it as ‘still needs to be validated’.
29. Uploading photos and linking these to the related data.
30. Being able to suggest changes in data via the virtual herbarium.
31. Making these suggestions for change visible for everyone.
32. Taking on the role of a validator by subscribing to everything that happens to specific species, specimens, etc. When someone makes a change to data that you are subscribed on, you should receive a notification.
33. Embargoing data or at least make it not yet available for external visitors of the virtual herbarium.
34. Downloading data sets in a usable format (MS-Excel).

## Ecological based scientists

### Needs for data

The ecological based scientists mentioned the following types of data, when they were asked which information they needed in order to fulfil their task scenarios.

1. Coordinates or location names of where the specimen was collected.
2. Uncertainty information about the conversion from place name to coordinates. How reliable are these coordinates? It should be indicated whether they were derived from the collector, or through geolocation from the locality name.
3. Information about the distribution of a species and, in the case of invasive species, it spread over time.
4. Date of collecting.
5. Date of observation (e.g. waarnemingen.be).
6. Number of individuals.
7. Timing of observations.
8. Frequency of observations.
9. Coordinates of observations.
10. Photographs of the observed specimen.
11. Information about the collector.
12. Current names and linked synonyms, vernacular names, local names, previous names.
13. Field notes that were added by the collector:
    1. Description of the morphological traits of a species
    2. Description of the circumstances in which the specimen was collected (flowering stage, colours, habitat, …)
14. Botanical drawings.
15. Abiotic factors of an environment.
    1. Clouds
    2. Weather
    3. Latitude
    4. Temperature
    5. Oxygen
    6. Salinity
    7. Soil (edaphic factors)
    8. Air
    9. Water
    10. Sunlight
    11. Humidity
    12. Topography
    13. pH
    14. Atmospheric gases
    15. Reproductive requirements of a species (what does it need to spread?)
16. Literature about:
    1. Distribution of species
    2. Habitat of species
    3. Climate change
    4. Invasive alien species reports
    5. Information on the interaction of species with other organisms
    6. Information on the interaction of species with other vegetation
    7. Human footprint
    8. Population density of a location
    9. Urbanization
    10. Human impact on the environment
    11. Anecdotes on the risks related to species

### Requirements of ecological based scientists

1. Using the virtual herbarium as a user-friendly filtering tool of the data that is present within the in-house herbarium catalogue.
2. Interactive visual search methods such as drawing an area on a map to look for a location or dragging a timeline to search a timeframe.
3. Adding extra filters to the results of a search query to look for relations between data (e.g. laying relations between distribution, time, and distribution of other species).
4. Filtering results based on collection date.
5. After searching and filtering the data we want to be able to download this data set in a usable format.
6. Generate a report based on the filtered data results.
7. Start the right nomenclature:
   1. Linking current names, synonyms, vernacular names, local names
   2. Using the interlinked nomenclature to find all relevant literature
   3. List of all synonyms related to a current name
8. Smart aggregation and consolidation of data:
9. Ecologists are fonder of lumping and like to start from consolidated data that gives a handy overview of the data results.
   1. For example, when looking for a collector they expect to get an overview of:
      1. Places where he/she collected visualized on an interactive map.
      2. Species that were collected by this collector.
      3. Published biographies of this collector.
      4. Literature related to his/her work.
   2. when looking on the level of species, they would like to see an overview of:
      1. The type specimen and protologue.
      2. Descriptions of the morphological traits.
      3. Literature related to this species.
      4. Overview of nomenclature (current, synonyms, vernacular, local).
      5. Distribution of the species based on collection coordinates of related specimens.
      6. Links to external databases that provide ecological, climatic data related to the collection location.
10. The more metadata the better
    1. High resolution images are valuable, as they often have to look at morphology of specimens.
    2. They focus more on data at the level of species, and are more likely to analyse specimen data aggregated, in comparison to taxonomists.
    3. All of the metadata on labels should be digitized.
    4. Location is very important for them, ideally coordinates.
11. Linking internal and external databases
    1. They strive to find links between data of a species, based on all of the metadata found on specimen level, and ecological, climatic information of a location.
    2. Linking the virtual herbarium of Botanic Garden Meise with the herbaria of other institutions would generate more metadata on the specimens and therefore more information at the level of species.
    3. Linking the internal databases would make it easier to get access to consolidated data (e.g. links to photographic material of living specimens when search for species)
12. Need for reliable and accurate data
    1. Clearly described origins of data.
    2. Seeing how coordinates were defined to make the accuracy of these coordinates clear.
    3. Being able to mark which collectors were known for fraudulent collecting of data.
    4. Consolidated overview of a collector would provide a map of places where this person collected specimens. This map would help validate specimens by showing where the collector could or could not have collected.

## Historical based scientists

### Needs for data

1. Administrational keywords.
2. Scientific keywords.
3. Bibliographic keywords.
4. Internal annotations.
5. Analytical annotations on material.
6. Bibliographic annotations.
7. Coordinates of location where the specimen was collected.
8. Name of the location where specimen was collected.
9. Collecting date of the specimen.
10. Information about the exchange of specimens.
11. Information about the source of data, for example, if the name of the collector was derived from the type of label used on the specimen, rather than their name being written on the specimen.
12. High resolution image of the original material.
13. Biographies of the collector.
14. Name of collector.
15. Portrait of collector.
16. Name of the identifier or determinator.
17. Bibliography of the collector.
18. Name of the owner of the original herbarium collection.

### Requirements of historical based scientists

1. Being able to reconstruct the origin of data.
2. Relating individual specimens back to their original collection.
3. New collections should be registered before the specimens get cut apart for further analysis
4. Being able to search by the determinator and herbarium owners, not just the person who collected the specimen.
5. Looking for relations between data.
   1. Creating links between internal and external databases:
      1. Library
      2. Herbarium
      3. Seed bank
      4. Living plant collection
      5. Photos of living plants
   2. Search functions should make it possible to search and filter on almost any type of metadata to look for possible relations and effects.
   3. System should provide smart links to other data that is somehow related to the data you are looking for.
   4. Linking current names, synonyms, local names, vernacular names, and previous names.
   5. Being able to see how the nomenclature of a specimen/species evolved over time (also related to data history).
   6. Who changed the name at what moment in time?
   7. By not only digitizing the name of the collectors, but also of the owners and those who identified the plants, it will be possible to look for links between these people.
   8. Which collectors worked together?
   9. How did collectors influence each other?
   10. How were collections passed on from owners to institutes?
   11. Which persons (collectors, identifiers, owners) had an influence on the metadata of a collection or a specimen?
6. More metadata to find more possible relations between data.
   1. Integrating data from external databases to get access to more metadata and create broader data sets.
   2. Easy access to a high resolution image of the original source of information. If we cannot digitize all data of the label of a specimen, it should be possible to view this on a high-resolution photo.
   3. Clear visual distinction between original and processed data. Processed data leads to a possible bias that might influence the further analysis of this data.
7. Using the virtual herbarium to not just access but also add data. System should be open enough to create new links between data, suggest changes and corrections of data, and add new data to the central database. Suggestion for the integration of citizen science. Everybody should be able to add new data or refine existing data.
8. Making it possible to access the portal anywhere at any time, so our African colleagues are free to add data as well without have to make the trip to Belgium.
9. The need for a less rigid database structure.
   1. The current database forces the users to merge their data within a rigid structure. This leads to a lot of bias and knowledge that gets lost in the process.
   2. The new virtual herbarium should be more flexible to avoid having to merge data in a prefixed structure.
10. Reliability
    1. Validation process of added data.
    2. If everyone would be able to add data, we will need a solid validation process.
    3. The validation process should be made more transparent. Requests for change should be visible and discussable for everyone.
    4. Data history. Change should not just overwrite the previous data, but should be added to the history of changes.
    5. Viewing a history of changes of data would make the data more reliable for other scientists, as you get insight into the how this data originated.
    6. After the search and filtering of data, it should be possible to download these results (data set) in a usable format for further analysis.
